# Supplementary material for: A theory for colors of strongly correlated electronic systems
Source: Nat Commun. 2023 Sep 9;14:5565. doi: 10.1038/s41467-023-41314-6 (PMC10492800; doi:10.1038/s41467-023-41314-6)
Supplement: Supplementary file 1 — Supplementary Information [file 41467_2023_41314_MOESM1_ESM.pdf]

A theory for colors of strongly correlated electronic systems

Swagata Acharya,[1,2] Dimitar Pashov,[3] Cedric Weber,[3] Mark van Schilfgaarde,[2] Alexander I. Lichtenstein,[4,5] and Mikhail I. Katsnelson[1]

[1]*Institute for Molecules and Materials, Radboud University, NL-6525 AJ Nijmegen, The Netherlands*
[2]*National Renewable Energy Laboratory, Golden, CO 80401, USA*[*]
[3]*King's College London, Theory and Simulation of Condensed Matter, The Strand, WC2R 2LS London, UK*
[4]*Institute of Theoretical Physics, University of Hamburg, 20355 Hamburg, Germany*
[5]*European X-Ray Free-Electron Laser Facility, Holzkoppel 4, 22869 Schenefeld, Germany*

SUPPLEMENTARY DISCUSSION

The supplemental materials discuss the Questaal implementation, band structural details from different levels of the theory, the role of higher order vertex corrections, incorporated in a many body perturbative approach, in leading a systematic improvement in quasi-particle description and collective charge excitations in these strongly correlated systems. It also discusses the paramagnetic band structures and the convergence of the imaginary part of the macroscopic dielectric response $\mathrm{Im}\,\epsilon$ with different sizes of the BSE Hamiltonian.

A detailed justification for why QS*GW* improves on conventional forms of 1-shot *GW* is described in §4 of Questaal's methods paper, Ref. [1] (heretofore referred to as Ref. I); in the paper describing Questaal's implementation of QS*GW*, Ref. [2] (heretofore referred to as Ref. II) and finally in Ref. [3], §IIC (heretofore refereed to as Ref. III). The enhancements DMFT brings when augmenting QS*GW* is presented in several works; see, e.g. (I, §5), and Refs. [4, 5].

Questaal Implementation of MBPT

Implementation of *GW* requires both a 1-body framework and a two-body framework. Both are described in detail I and II. I places heavier focus on the one-body part, while II focuses on the *GW* theory and its implementation. Ref. III explains how ladder diagrams are implemented in both the polarizability and self-energy in many-body perturbation theory, and provides extensive benchmarks of results.

Questaal is an all-electron method, with an augmented wave basis consisting of partial waves inside augmentation spheres, constructed from numerical solutions of the radial Schrodinger equation on a logarithmic mesh (I, §2.2). The one-body basis consists of a linear combination of smooth, atom-centered Hankel functions as envelope functions, augmented by the partial waves. Two partial waves are calculated at some linearization energy ϕ_ℓ and energy derivative $\dot{\phi}_\ell$, which provides enough freedom to match value and slope to the envelope functions (I, §3).

One particle basis: In a conventional LMTO basis, envelope functions consists of ordinary Hankel functions, parameterized by energy E. Questaal's smooth Hankel functions are composed of a convolution of Gaussian functions of smoothing radius r_s, and ordinary Hankel functions (I, §3.1); thus two parameters are needed to define the envelope. In the periodic solid, Bloch sums of these functions are taken (I, Appendix C). In the present work, E is constrained to a fixed value (-0.4 Ry), and r_s determined by optimizing the total energy of the free-atomic wave function. These are kept fixed throughout the calculation, while the partial waves and linearization energy float as the potential evolves. By fixing E to a universal value, we are able to take advantage of the "screening transformation" to render the basis set short-ranged (see I, §2.9). This can be useful for the interpolation of the self-energy to an arbitrary k mesh, as described below. A second envelope function of a deeper energy is needed to make the hamiltonian reasonably complete. The latter energy is chosen to be 0.8 Ry deeper than the first. The envelopes of orbitals l=0. . .4 are employed the first energy (25 orbitals), and l=0. . .3 for the second. At the *GW* level, a few other additions are made to make the basis more complete. To expand the hilbert space inside the augmentation spheres, a local orbital ϕ_z may be added (I, §3.7.3). ϕ_z is a solution of the radial Schrodinger equation at an energy, either well below the linearization energy for deep core-like states, or well above it to better represent the unoccupied states. In the present work we used the Ni and Mn 4d local orbital.

k convergence: The GW mesh and the one-body mesh are generally different: the latter normally needs to be somewhat finer, as the self-energy is a relatively smooth function of k while the kinetic energy is less so. Since the cost is low, we use a finer mesh than necessary for the one-particle part, which obviates the need to test the mesh for k convergence. Careful tests of the GW mesh were made for each system. For the simple AFM structures we used a $4{\times}4{\times}4$ mesh for the self-energy, and an $12{\times}12{\times}12$ mesh for the one-particle part. for the PM structures (volume increased by 8-fold) the meshes were reduced to $2{\times}2{\times}2$ and $4{\times}4{\times}4$.

To enable inequivalent meshes, the self-energy must be interpolated. To render the interpolation everywhere smooth, (I, §2G) eigenfunctions and self-energy are rotated to the LDA basis, and the full self-energy matrix is kept only up to a cutoff above the Fermi level in this basis, denoted $\Sigma_{\rm cut}$ in the Table. Above this cutoff, only the diagonal part of Σ is kept. $\Sigma_{\rm cut}$ may be made arbitrarily high, but if it is too high the interpolation is no longer smooth. Fortunately the result depends weakly on $\Sigma_{\rm cut}$. In this work we used $\Sigma_{\rm cut}$=2.5 Ry.

A smooth Hankel functions has a plane-wave representation; thus any linear combination of them, e.g., an eigenfunction, does also. An eigenfunction represented in this form is equivalent to a representation in an LAPW basis: it is defined by the coefficients to the plane waves, the shape of the partial waves and their coefficients (which are constrained to match smoothly onto the envelope functions). We used 6.2 Ry as PW cutoff for the one-particle basis.

Two-particle basis: The two-particle basis is needed to represent quantities such as the bare coulomb interaction and the polarizability. As with the one-particle basis, it as a mixed construction with interstitial parts and augmentation parts (II, §IIA):

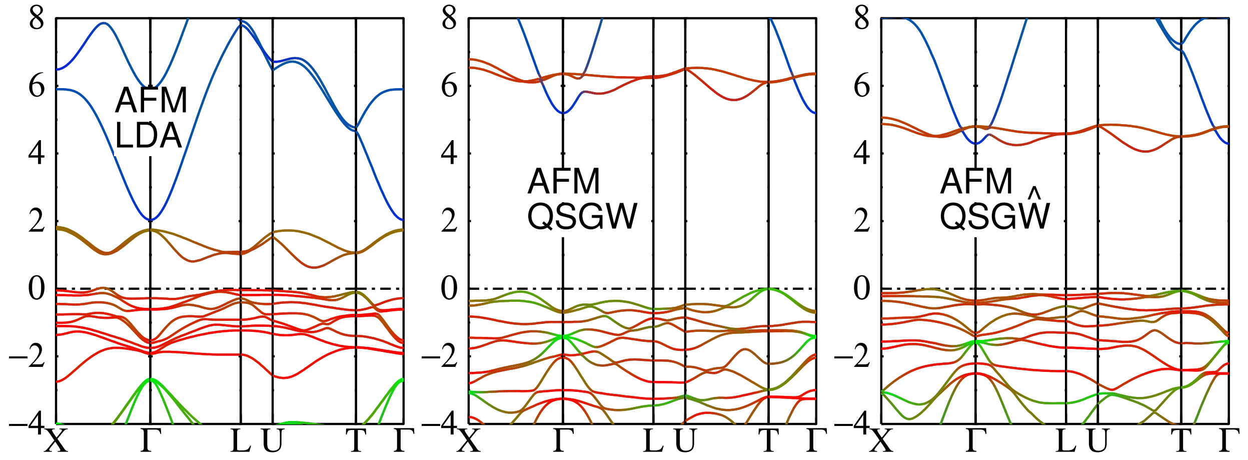

Supplementary Figure 1. **Electronic band structure of NiO**: Top panels show energy band structures for antiferromagnetic NiO, in the local-density, QSGW, and QS$G\widehat{W}$ approximations. Blue and red correspond to Ni *sp* and *d* character, respectively; green to O character. The electronic gap is 0.5 eV in LDA, 5 eV in QSGW and 4.0 eV in QS$G\widehat{W}$.

envelope function products are represented as plane waves, since product of plane waves is another plane wave. Thus the interstitial parts of the mixed (product) basis are plane waves, with its own PW cutoff. We used 5.0 Ry as the cutoff. Inside augmentation spheres, all possible products of partial waves are called product functions B_ℓ, organized by ℓ with a form $B_I{=}B_\ell(r)Y_{\ell m}(\hat{\mathbf{r}})$. The set of all possible products of partial waves is somewhat overcomplete with a relatively large rank. It is reduced by diagonalizing the overlap matrix, and retaining the subset of functions above a cutoff eigenvalue of the overlap. It has been found from experience that eigenfunctions with eigenvalues below $3{\times}10^{-4}$ for ℓ=0,1 and 10^{-3} for $\ell{>}1$ have essentially negligible effect on the result, and are discarded. The product basis is truncated at a finite ℓ; we used $\ell_{\rm cut}{=}8$ in this work.

GW: Bare coulomb interaction: To stabilize the calculation, the bare coulomb interaction, $v(q) = 1/q^2$, is approximated by a Thomas-Fermi form, $v(q) = 1/(q^2 + V_{\rm TF})$. This is because if $V_{\rm TF}$ is set to zero, the result can become unstable. We use a small value $V_{\rm TF}$, typically $2{\times}10^{-5}$ Ry, though sometimes somewhat larger values, up to $2{\times}10^{-4}$ Ry were used. The dielectric constant, ϵ_∞, can vary by a few percent over this range. For that reason ϵ_∞ was calculated for several values of $+V_{\rm TF}$, e.g. $1{\times}10^{-5}$, $1{\times}10^{-5}$, and $3{\times}10^{-5}$ Ry, and the reported value is the result when extrapolated to zero.

Frequency mesh: to construct the self-energy, an energy integration on the real frequency axis is taken. A regular quadratic mesh of the form $\omega_i = \texttt{dw}{\times}i + \texttt{dw}^2 i^2/(2\omega_c)$ is used, with i spanning ω_i=0 and the largest eigenstate. Points are linearly spaced for $\texttt{dw} \ll \omega_c$, but the spacing increases for $\texttt{dw} \gtrsim \omega_c$. It has been found empirically that results are essentially independent of mesh for $\texttt{dw}{<}0.08$ Ry and $\omega_c{\gtrsim}0.1$ Ry. In practice we use $\texttt{dw}{=}0.02$ Ry and $\omega_c{=}0.2$ Ry to obviate the need for checking convergence. To pick up the poles of G and W to make Σ, the contour is deformed to include an integration on the imaginary axis of ω (I, §2F). In all the calculations used here, we used 6 points on a Legendre quadrature. A few checks showed that the result hardly depended on the number of points in the quadrature.

Manual vs auto-generated input: Questaal has an automatic generator, `blm`, to construct input files from structural data. Most input parameters are automatically generated by `blm`, such as the MT radii $r_{\rm MT}$, the product basis cutoffs, and the plane wave cutoffs, the Gaussian smoothing radius defining the envelope functions, and the placements for floating orbitals, when they are sought. Also for the vast majority of parameters, the code uses default values if inputs are not explicitly specified. For a few parameters, manual intervention is needed to monitor convergence, especially the number of k points and the plane wave cutoffs. Hankel function energies E must be manually set, but usually fixed values noted above are sufficient. Results are largely insensitive to the choice of E, provided it is not pushed too deep.

NiO: $p-d$ alignments and band gaps at different levels of the theory

Supplementary Fig. 1 compares three levels of theory for the band structure of NiO. The self-energy corrections in QSGW significantly modify the $p{-}d$ alignment and also the $t_{2g}{-}e_g$ alignment relative to the LDA. In the LDA the splitting between occupied and unoccupied d levels is severely underestimated, and the O 2p is severely misaligned with the Ni 3d states. QSGW tends to overestimate the $d{-}d$ splitting (screening is too small, making the potential too close to Hartree Fock), so the electronic band gap within QSGW remains about 20% too high compared to experimental observations. QS$G\widehat{W}$ shifts Ni-*sp* states by

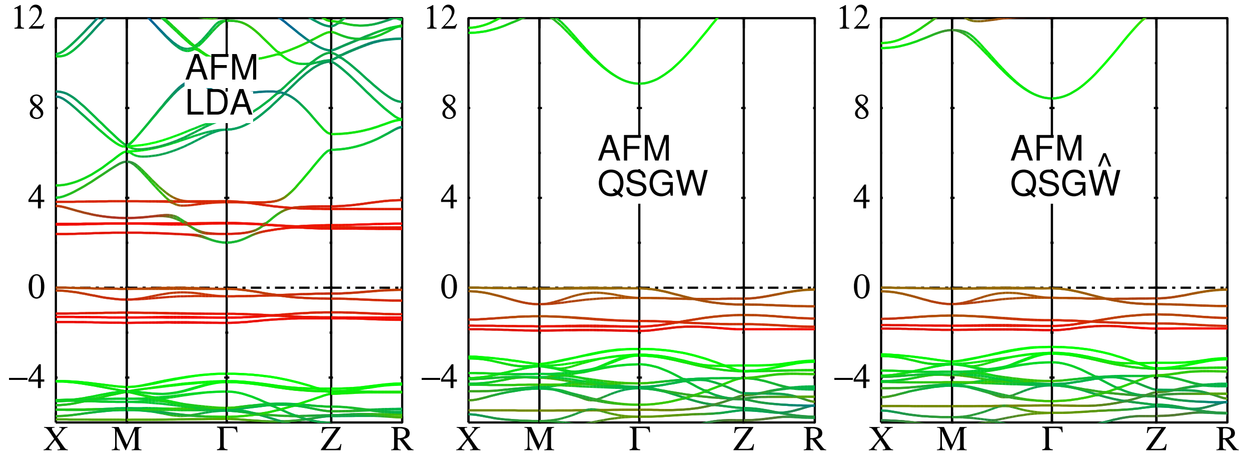

Supplementary Figure 2. **Electronic band structure of $\mathbf{MnF_2}$**, LDA, QSGW and QS$\widehat{GW}$. Red and green correspond to Mn d (majority spin in the valence band and minority spin in the conduction band), and O p character. The electronic gap is 2.0 eV in LDA, 9.1 eV in QSGW and 8.4 eV in QS$\widehat{GW}$. (Had local orbitals been included for the F 3s and F 3p states, the gap would decrease lightly, by about 0.2 eV.) QSGW (QS$\widehat{GW}$) minority d bands lie above the figure, at around 14 eV (12 eV).

0.7 eV and also the Ni-e_g, which leads to a reduction of the QSGW gap by ~1.0 eV. It is important to note the BSE corrects the electronic eigenfunctions from QSGW in an orbital dependent manner.

$\mathbf{MnF_2}$: LDA, QSGW and QS$\widehat{GW}$ compared

MnF_2 provides an extreme instance of errors incurred by the LDA (Supplementary Fig. 2). The bandgap (2.0 eV) is very small and the splitting between occupied and unoccupied d levels is severely underestimated. Also, the O(2p)-Mn(3d) splitting is much greater than in QSGW. The severe limitations of DFT and also HSE in case of MnF_2 were discussed in a previous work [6]. QS$\widehat{GW}$ reduces the QSGW gap by 0.7 eV.

NiO and $\mathbf{MnF_2}$: antiferromagnetic and paramagnetic solutions within QSGW

We simulate the fully ordered antiferromagnetic and disordered paramagnetic phases of NiO and MnF_2 and compute the macroscopic dielectric response in both the cases. A 2×2×2 superlattice of the AFM phase is formed (16 Ni or Mn atoms). To simulate the PM phase, spins are disordered in a quasirandom fashion [7] so that the shortest and second shorted pair correlation functions and three-body correlation functions are equivalent to random configurations. Supplementary Fig. 3 compares the AFM energy band structure (in the 2×2×2 superlattice) to the PM for NiO (top panels) and MnF_2 (bottom panels). For NiO, the main change in the AFM→PM transition is a difference in the O(2p)-Ni(3d) alignment. Note how in the PM case the O p states are pushed up to yield a greater amount of O 2p character in the upper valence bands. There is also a reduction of ~0.2 eV in the bandgap. The AFM→PM change is minor in MnF_2: the gap reduces by ~0.5 eV.

Excitonic abosption energies (eV)	Oscillator strength without SOC	Oscillator strength with SOC
1.6	10^{-10}	10^{-7}
3.6	15.8	15.8

TABLE I. **Effect of SOC on the oscillator strength of the dark excitons**. SOC enhances the oscillator strength of the 1.6 eV exciton and has no impact of the 3.6 eV optical shoulder. However, the 1.6 eV exciton still remains dark and at least two orders darker than the darkest ecitons from CrI_3

The AFM→PM difference is associated with the magnetic ordering energy scales in respective materials. We observe emergence of several bright excitons in both NiO and MnF_2 in the paramagnetic phase. However, in NiO, an otherwise dark exciton in the visible range from the AFM case, becomes bright in the paramagnetic phase while in MnF_2 no new excitons emerge in the visible range when the system is magnetically disordered.

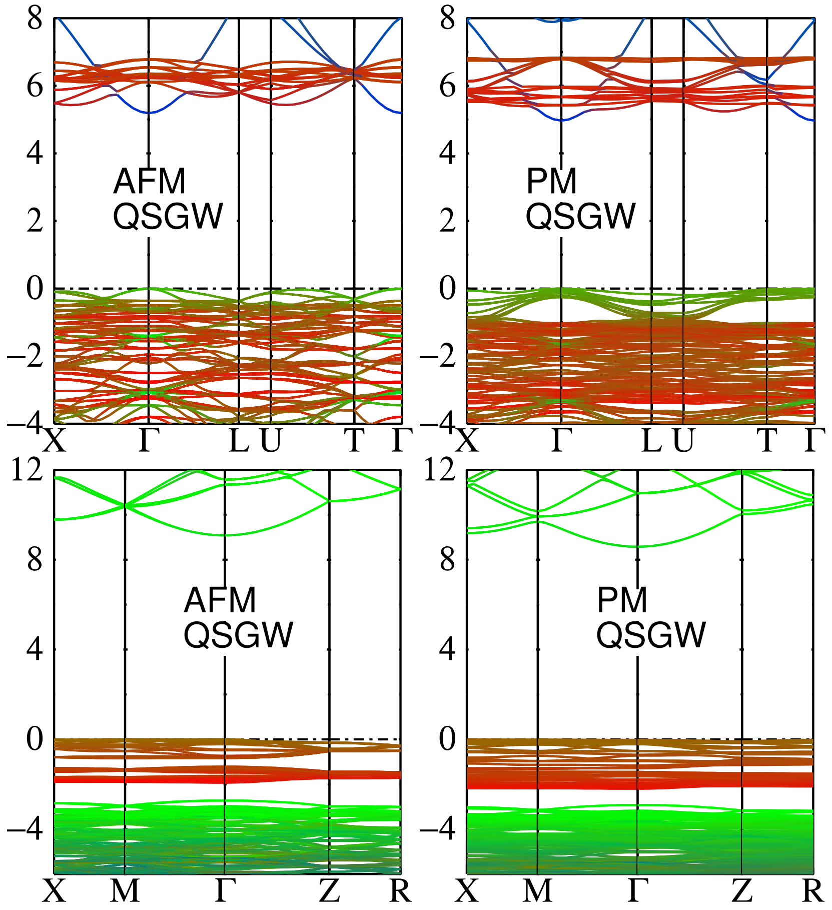

Supplementary Figure 3. **AFM and paramagnetic phases, Energy bands for NiO and MnF_2** : Top panels compare antiferromagnetic NiO in a 2×2×2 supercell, and paramagnetic NiO in the same cell with quasirandom spin arrangements. Bottom panels are the same for MnF_2. The AFM results are equivalent the QS*GW* calculations of Figs. 1 and 2, with the Brillouin zone folded. The red and green colors correspond to the projections of one-particle eigenstates onto $3d$ metal (Ni, Mn) and ligand (O,F) respectively.

Supplementary Fig. 4 shows convergence in excitonic peak positions by solving different sizes of the BSE Hamiltonian. Beyond 64 valence and 64 conduction bands (included in the BSE Hamiltonian) the eigenvalues stops changing. With 2×2×2 k-mesh and v=64 and c=64, this amounts to solving a BSE eigenvalue problem with matrix of rank 8×64×64=32768.

Finally, we show the irrelevance of SOC in determining the green color of NiO. The Supplementary table I shows the oscillator strengths of the relevant excitonic absorptions with and without SOC. Supplementary Figure 5 shows the macroscopic dielectric response computed with and without SOC.

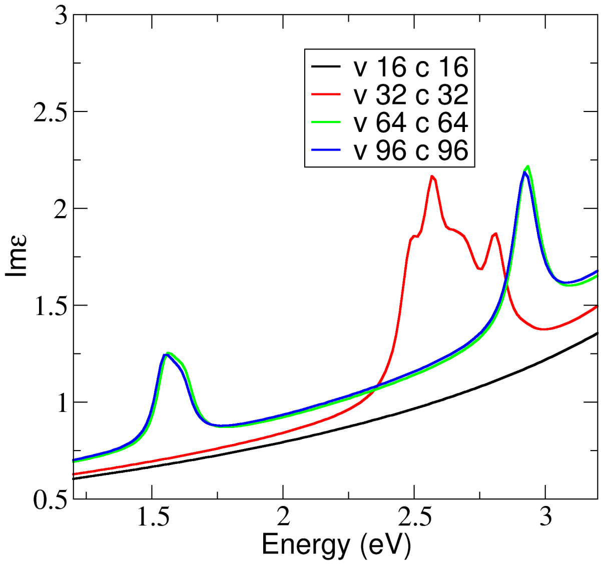

Supplementary Figure 4. **Convergence in exciton eigenvalues with sizes of the BSE Hamiltonian in paramagnetic NiO** : We show the convergence in the exciton eigenvalues in the paramagnetic QS$G\widehat{W}$ simulation of NiO. We see that inclusion of 64 bands from the valence (v) and conduction (c) allows us to converge two excitonic eigenvalues at 1.6 eV and 2.8 eV simultaneously.

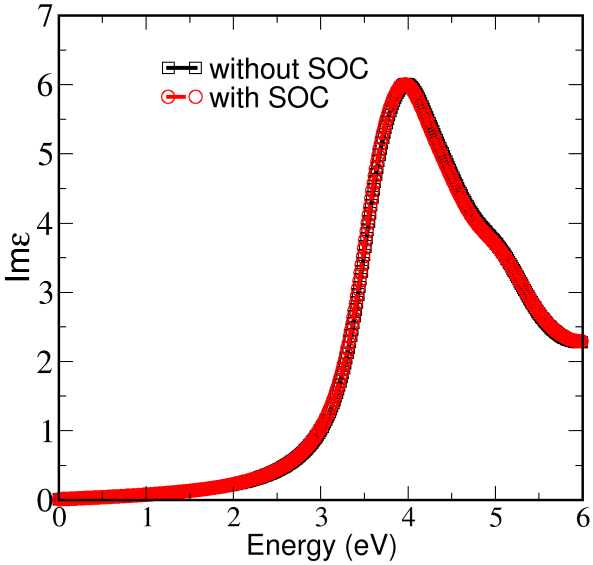

Supplementary Figure 5. **Optical absorption with and without spin-orbit coupling** : Inclusion of SOC has no effect on the overall structure of the optical absoption in NiO. It does impact the oscillator strengths of the dark excitons but they reman dark even with the inclusion of SOC.

* swagata.acharya@nrel.gov

[1] D. Pashov, S. Acharya, W. R. L. Lambrecht, J. Jackson, K. D. Belashchenko, A. Chantis, F. Jamet, and M. van Schilfgaarde, "Questaal: a package of electronic structure methods based on the linear muffin-tin orbital technique," *Comp. Phys. Comm.*, vol. 249, p. 107065, 2020.

[2] T. Kotani, M. van Schilfgaarde, and S. V. Faleev, "Quasiparticle self-consistent *GW* method: A basis for the independent-particle approximation," *Phys. Rev. B*, vol. 76, p. 165106, 2007.

[3] B. Cunningham, M. Grüning, D. Pashov, and M. van Schilfgaarde, "Qsgw: Quasiparticle self consistent gw with ladder diagrams in w,", *ArXiv 2302.06325*, 2023.

[4] S. Acharya, D. Pashov, E. Chachkarova, M. van Schilfgaarde, and C. Weber, "Electronic structure correspondence of singlet-triplet scale separation in strained Sr_2RuO_4," *Appl. Sci.*, vol. 11, p. 508, 2021.

[5] S. Acharya, D. Pashov, and M. van Schilfgaarde *Phys. Rev. B*, vol. 105, p. 144507, 2022.

[6] P. Das and T. Bazhirov, "Electronic properties of binary compounds with high fidelity and high throughput," in *Journal of Physics: Conference Series*, vol. 1290, p. 012011, IOP Publishing, 2019.

[7] A. Zunger, S.-H. Wei, L. G. Ferreira, and J. E. Bernard, "Special quasirandom structures," *Phys. Rev. Lett.*, vol. 65, p. 353, 1990.
